# Colo-enteric fistula associated with diffuse large B cell lymphoma that resulted in gastrointestinal bleeding: A case report and literature review

**DOI:** 10.1016/j.ijscr.2022.106798

**Published:** 2022-01-31

**Authors:** Shih-Wei Chiang, Tzu-Wei Chiang

**Affiliations:** a Division of Colorectal Surgery, Taiwan, ROC; b Department of Surgery, Taichung Veterans General Hospital Chiayi Branch, Chiayi City, Taiwan, ROC; c Department of Surgery, Taichung Veterans General Hospital, Taichung City, Taiwan, ROC

**Keywords:** NHL, non-Hodgkin lymphoma, GI, gastrointestinal, CT, computed tomography, TAE, transarterial embolization, SMA, superior mesenteric artery, MALT, mucosa-associated lymphoid tissue, DLBCL, diffuse large B-cell lymphoma, EATL, enteropathy-associated T-cell lymphoma, MCL, mantle cell lymphoma, FL, follicular lymphoma, MLP, multiple lymphomatous polyposis, CTE, computed tomography enterography, Case report, Primary gastrointestinal lymphoma, Colo-enteric fistula, Gastrointestinal bleeding

## Abstract

**Introduction and importance:**

The most common symptoms of primary gastrointestinal (GI) lymphoma are non-specific, such as nausea, vomiting, diarrhea, weight loss, and abdominal pain. The rare acute symptoms include bowel obstruction, intussusception, and perforation. Primary small bowel lymphoma accounts for the smallest proportion of all GI malignancies. We report a case of intestinal lymphoma presenting with bloody stools and anemia.

**Case presentation:**

The patient initially underwent both duodenoscopy and colonoscopy with negative findings. Isotopic red blood cell (RBC) scan was then performed due to persistent bleeding along with computed tomography angiography (CTA) because of suspected bleeding in the left abdomen. Successful embolization over the arcade of the sigmoid and left colic arteries was performed. However, the bleeding did not stop, and ischemic colitis was diagnosed by repeat colonoscopy. A coloenteric fistula was finally discovered during emergent laparotomy.

**Clinical discussion:**

GI lymphomas are a rare disease entity among the all GI malignancies. Despite acute abdominal symptoms including obstruction, perforation, bleeding and intussusception, enteral fistula is also one of the complications. It was seen to be a long-term complication after treatment or disease process in most of cases, however it could occur as the initial manifestation. GI bleeding is a life-threatening condition and commonly needs prompt decision making. There were no standard managements for these patients, it depends on clinical judgements from physician individually.

**Conclusion:**

This is a rare condition that has not been previously described in Taiwan. Early diagnosis and timely management will decrease morbidity and mortality in the GI lymphoma population.

## Highlights

• Gastrointestinal (GI) lymphomas is a rare disease entity among the all GI malignancies.
• Despite acute abdominal symptoms including obstruction, perforation, bleeding and intussusception, enteral fistula is also one of the complications.
• Early diagnosis and timely management will decrease morbidity and mortality

## 1 Introduction

The gastrointestinal (GI) tract is the most common extranodal site in non-Hodgkin lymphoma (NHL) [1], accounting for 5% to 20% of all cases. However, primary intestinal lymphoma is relatively rare, accounting for only 1%–4% of cases [2]. The clinical symptoms may be related to the localized disease, including abdominal pain, nausea, vomiting, diarrhea, and malabsorption. GI bleeding, perforation, and intestinal obstruction are infrequent. Considering the rarity and non-specific symptoms in most patients with primary intestinal lymphoma, it is difficult to establish an early definite diagnosis, and prognosis is usually poor when diagnosed at an advanced stage.

GI bleeding is a common clinical symptom encountered by general surgeons and gastroenterologists [3]. Patients may be categorized as stable or unstable to identify the subsequent algorithm for bleeding localization and control. Isotopic red blood cell (RBC) scan and computed tomography (CT) angiography are well-described diagnostic modalities for GI bleeding, and CT angiography reportedly has a higher accuracy in localization [4]. Surgical indications include unapproachable angiography in unstable patients, recurrent bleeding despite repeated endoscopic intervention in stable patients, or continuous bleeding for 72 h.

Here, we report a case of lymphoma with coloenteric fistula formation, which resulted in massive GI bleeding. This case report has been reported in line with the SCARE 2020 criteria [5].

## 2 Presentation of case

A 64-year-old man presented to our emergency room (ER) with exertional dyspnea and tarry stools for 2 weeks. He gave a past medical history of hypertension and diabetes mellitus. He also underwent a Billroth II subtotal gastrectomy 30 years ago for peptic ulcer perforation.

The patient reported black stools, mild upper abdominal fullness, and poor appetite for the last 2 months. He underwent temporary hospitalization at another hospital, where he received upper and lower GI endoscopies as well as abdominal CT scan but there were no obvious findings. The patient was discharged after symptomatic treatment. However, he complained of shortness of breath and dizziness later. Hence, he was brought to the ER, where a blood test showed anemia (hemoglobin [Hb] = 7.8 g/dL). Packed red blood cells were transfused, but there was no improvement and the hemoglobin continued to decrease. Suspecting gastrointestinal (GI) bleeding, the patient was transferred to the GI ward for further management.

Upper and lower GI endoscopies were arranged for identifying possible bleeding sites. There was no bleeding from the esophagus, stomach, or duodenum in upper GI endoscopy. Lower GI endoscopy was performed up to the cecum, and no bleeders or mucosal lesions were detected. An isotopic RBC scan was then arranged, which revealed increased tracer accumulation in the left abdomen. Since small intestinal bleeding was suspected, we arranged double balloon enteroscopy; however, it failed because of the presence of a large amount of bloody stool; hence, computed tomography angiography (CTA) was performed.

**Fig. 1:**
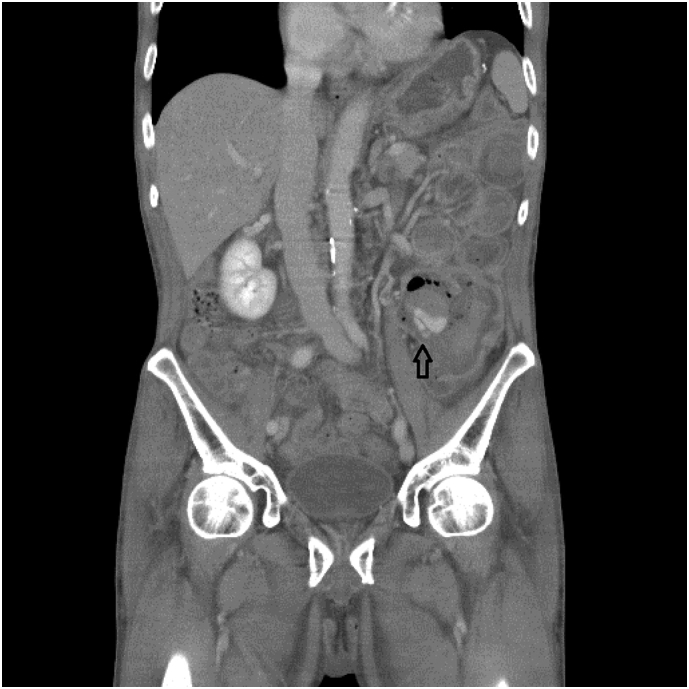
Contrast extravasation in the sigmoid colon (*Arrow*).

**Fig. 2:**
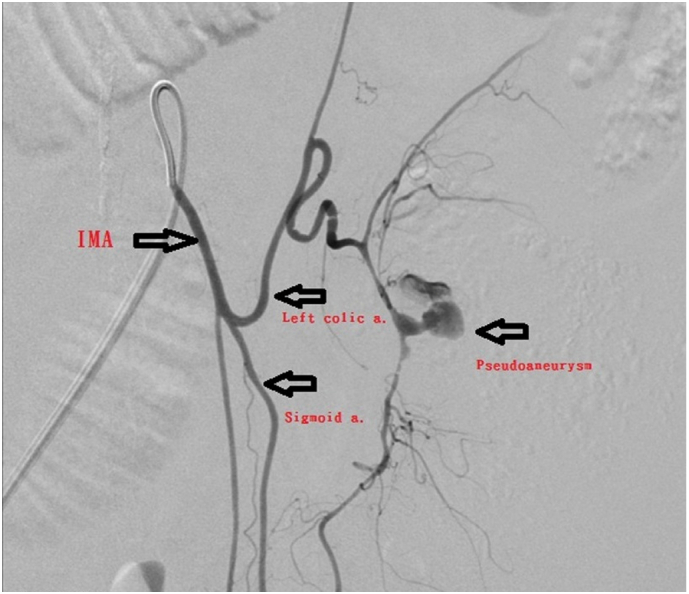
Angiography showing pseudoaneurysm at the arcade of the left colic artery and sigmoid artery (IMA: Inferior mesenteric artery).

**Fig. 3:**
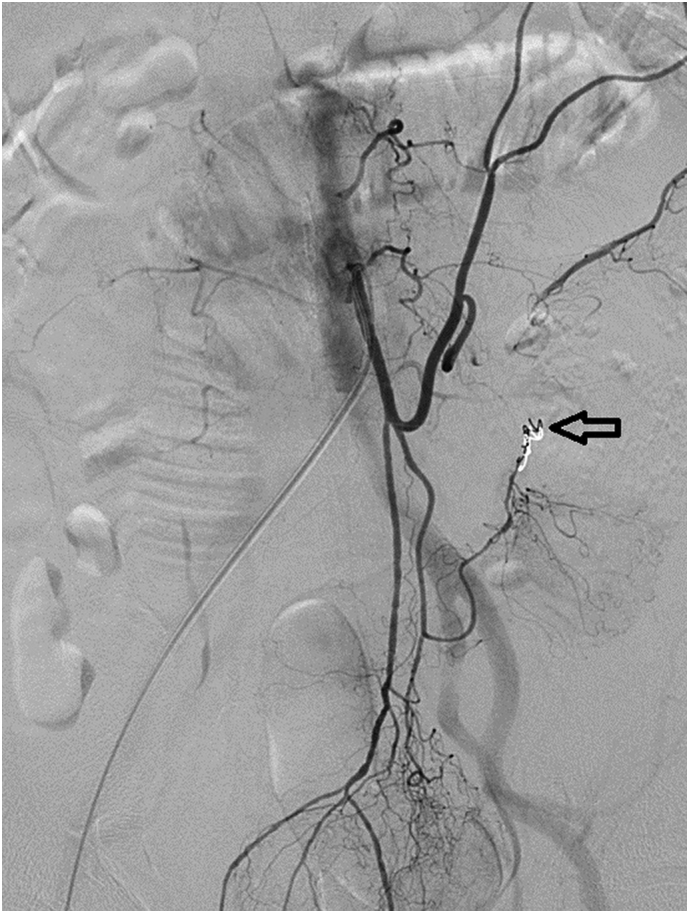
Pseudoaneurysm was occulted with coils and gel foam cubes (*Arrow*).

**Fig. 4:**
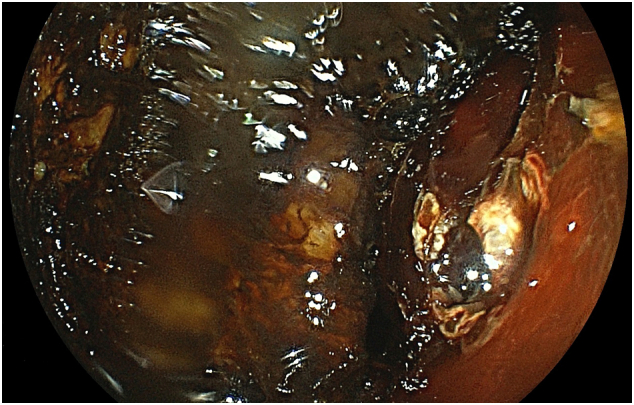
Lower gastrointestinal (LGI) endoscopy showing a diffuse circumferential ulcer with active oozing in the colon.

**Fig. 5:**
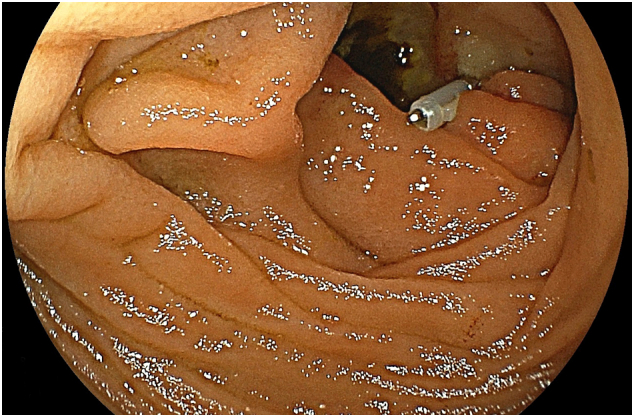
Lower gastrointestinal (LGI) endoscopy showing the ulcer localized with a hemoclip.

The angiographic report showed a pseudoaneurysm over the arcade between the left colic artery and sigmoid artery with prominent extravasation (Fig. 1). We consulted interventional radiologists for emergency transarterial embolization (TAE). They inserted spring coils and gel foam cubes into the pseudoaneurysm (Fig. 2, Fig. 3). No contrast extravasation was observed at the end. The superior mesenteric artery (SMA) series was also checked without evidence of bleeding. However, the patient still complained of bloody stools after TAE. The follow-up hematocrit continuously dropped despite transfusion with 6 units of packed RBCs in the last 24 h. A second-look colonoscopy revealed a diffuse circumferential ulcer with active oozing at the sigmoid colon (Fig. 4). Ischemic colitis, Crohn's disease, or infectious colitis was considered as potential causes. Hemoclip was applied for localization, and a colorectal surgeon was consulted for surgical exploration (Fig. 5).

**Fig. 6:**
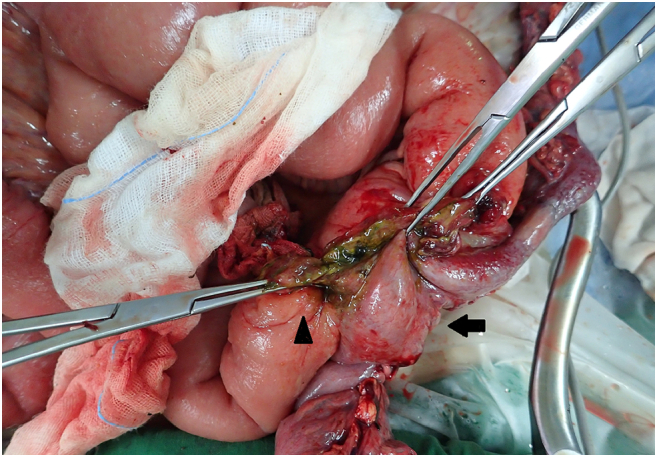
Operative findings demonstrated a colo-enteric fistula (*Arrow*: sigmoid colon; *Arrowhead*: small bowel).

An emergency laparotomy was performed after obtaining informed consent. The operative findings showed a fistula between the sigmoid colon and ileum, and abscess formation beside the fistula (Fig. 6). Hence, the patient underwent segmental resection of the small bowel and subtotal colectomy with ileocolic anastomosis. The resected specimen revealed an ulcerative lesion over the ileum and an adhesion between the distal part of the colon and ileum. The hemoclip was discovered in the ileal mucosa after reviewing the resected fistula, which was previously considered a marking point in the colonic mucosa. It was compatible with a coloenteric fistula. The final pathological diagnosis was diffuse large B-cell lymphoma involving the colon and small intestine. The patient recovered gradually after postoperative care and total parenteral nutrition support. Hematocrit also increased to 10.4 g/dL and no GI bleeding was reported after surgery. The postoperative course was smooth, and the patient was discharged in a stable condition. We will be followed up in the hematology clinic for further adjuvant chemotherapy.

## 3 Discussion

GI lymphomas account for 1%–4% of GI malignancies, 10%–15% of NHLs, and 30%–40% of extranodal NHLs [6]. The most common site is the stomach, followed by the small intestine and colorectum [7]. According to a study, the frequently involved sites in primary GI lymphomas are the stomach (65%), small intestine (20%–30%), colon (10%–20%), and esophagus (<1%) [8].

Primary small intestinal lymphomas differ in the histological features and genetic factors, and include mucosa-associated lymphoid tissue (MALT) lymphoma, diffuse large B-cell lymphoma (DLBCL), enteropathy-associated T-cell lymphoma (EATL), mantle cell lymphoma (MCL), follicular lymphoma (FL), and immunoproliferative lymphoma [9]. MALT lymphoma is associated with *Helicobacter pylori* infection and is predominantly found in the stomach. Patients with *H. pylori* gastritis are at increased risk of developing gastric MALT lymphoma, but the small bowel is rarely the primary site [10]. DLBCL can be categorized into three groups: germinal-center B-cell-like, activated B-cell like, and primary mediastinal DCBCL according to the gene expression patterns [11]. The current and widely accepted management of intestinal DLBCL involves surgical resection followed by chemotherapy. The chemotherapy regimens consisted of cyclophosphamide, doxorubicin, vincristine, and prednisolone (CHOP), or rituximab plus CHOP [12]. EATL is a rare and rapidly fatal type of intestinal T-cell NHL. The advisable treatment is local debulking in the early stage, and anthracycline-based chemotherapy in the advanced stage [13]. A large multicenter cohort study [14] showed poor prognosis of EATL, with a 5-year overall survival of only 11%. Cyclin D1 is the specific marker of MCL, which is produced by the tumor cells due to a t(11;14) translocation that results in overexpression [15]. It can specifically present with involvement of tonsils and the Waldeyer's ring, which accounts for 25% of patients [15]. Multiple lymphomatous polyposis (MLP) is the most common GI presentation of MCL. Numerous polyps are present throughout the GI tract, although MLP commonly occurs in the ascending colon and ileum [16]. GI-FL is a rare disease that accounted for only 3%–4% of all GI-NHL cases in a previous study [17]. FL is a low-grade lymphoma with slow development. The standard treatment is not well-established, but includes chemotherapy, radiotherapy, monoclonal antibody therapy, or a combination of the above therapies [18].

The common clinical symptoms of GI lymphomas are non-specific, such as nausea, vomiting, weight loss, abdominal pain, and rarely, acute abdominal symptoms (obstruction, perforation, bleeding, or intussusception) [19]. Enteral fistula is also one of the complications; it occurs mainly after surgery or chemotherapy [20]. Very few studies have reported enteral fistulas as the initial manifestation [20]. Wang et al. described an elderly patient with chronic diarrhea, abdominal cramping pain, and weight loss, who was diagnosed with DCBCL with enteric colic fistula [21]. Zhuang et al. reported two cases of GI-NHL with ileal-sigmoid fistulas diagnosed by computed tomography enterography (CTE); both patients complained of abdominal pain and chronic diarrhea [22]. Chun et al. reported a T-cell lymphoma with a jejuno-colic fistula in a patient who underwent colonoscopy due to diarrhea and abdominal pain [23].

Entero-colonic fistulas are usually found with inflammatory diseases, such as inflammatory bowel disease. They can also be caused by diverticulitis, abdominal surgery, foreign bodies, and malignancy. A fistula is considered a chronic and long-term process [22]. Generally, the tumors are located in the submucosa in the early stage of intestinal lymphoma, and then grow and invade the serosa and penetrate into the mesentery, resulting in an abscess or intestinal fistula after the lesions infiltrate the tissues adjacent to the intestinal wall [24]. Our patient had persistent GI bleeding as the initial presentation and underwent endoscopies with negative results. The bleeder was identified by an isotopic RBC scan, and he subsequently underwent successful TAE over the colonic peripheral branch supply. However, due to persistent GI bleeding with decreasing hematocrit level, we hypothesized that the bleeding may be from a tumor over the intestinal mucosa or ruptured colonic mucosa after TAE. This scenario is a rare complication of enteral fistula in primary GI lymphomas.

GI bleeding is a rare but life-threatening condition in 2.2%–22% of small and large bowel lymphomas [25]. There are no reports on the prognosis or overall survival of these patients. Furthermore, since emergency presentations are uncommon, it is difficult to acquire a sufficient number of cases for study. The evolving classifications of histological features and complex management make it difficult to compare the results in previously reported literature.

## 4 Conclusion

We encountered an unusual presentation of a rare disease entity, for which there was no standard algorithm for management. We hope that further studies will provide additional evidence to develop treatment guidelines or protocols for patients presenting with similar surgical emergencies.

## Provenance and peer review

Not commissioned, externally peer-reviewed.

## Source of funding

None.

## Ethical approval

I declare on my honor that the ethical approval has been exempted by my establishment.

## Consent

The patient provided informed consent, and the study design was approved by the appropriate ethics review board.

## Author contribution

Dr. Tzu-Wei Chiang: Corresponding author, writing the paper.

## Research registration

None.

## Guarantor

Dr. Feng-Fan Chiang.

## Declaration of competing interest

The authors confirm that there are no conflicts of interest.
